# Preparation and Characterization of Highland Barley Distillers’ Grains Gliadin–Chitosan Nanoparticles and Composite Properties

**DOI:** 10.3390/molecules30163390

**Published:** 2025-08-15

**Authors:** Qian Lv, Yiquan Zhang

**Affiliations:** College of Agriculture and Animal Husbandry, Qinghai University, Xining 810016, China; 15297018607@163.com

**Keywords:** gliadin from highland barley distillers’ grains, chitosan, nanoparticles, property characterization, stability

## Abstract

In embedding systems, protein–polysaccharide complexes can be utilized as wall materials to improve the bioavailability and activity of bioactive substances during delivery. This study used the antisolvent precipitation method to manufacture gliadin from highland barley distillers’ grains (HBDGG)–chitosan (Cs) nanoparticles. Using a variety of characterization techniques, the microstructure and interaction mechanism of HBDGG-Cs nanoparticles were examined, and their stability was assessed. In comparison to HBDGG, the results indicated that the addition of Cs enhanced the intensity of UV absorption and reduced the intensity of fluorescence. The content of α-helix dropped, while β-sheet, β-turn, and irregularly coiled content rose in the complexes. Hydrogen bonding, hydrophobic interactions, and electrostatic interactions were the primary forces that formed the nanoparticles. The contact force between HBDGG and Cs enhanced the stability of the nanoparticles. The particle size, polydispersity index (PDI), and zeta potential were 526.10 ± 11.78 nm, 0.20 ± 0.06, and 51.31 ± 0.66 mV, respectively, at a mass ratio of 1:1 between HBDGG and Cs. The nanoparticles exhibited good ionic, acid-base, and storage stability in addition to being widely distributed. This work offers a theoretical foundation for employing HBDGG-Cs nanoparticles to deliver bioactive components in food as well as a novel method for the comprehensive usage of HBDGG and Cs.

## 1. Introduction

Highland barley is an annual herbaceous grass that is mostly grown in Tibet and Qinghai, China. It is a member of the barley genus. It is also referred to as naked barley since its seeds are visible [1]. Rich in vitamins, dietary fiber, protein, and other useful ingredients, barley is incredibly nutrient-dense [2]. The protein level of highland barley is higher than that of rice, wheat, corn, and other cereals [3], with an average of 12.43% and a range of 6.35% to 23.40%. This comprises gliadin, glutenin, serum, and globulin [4]. The leftover residue from making barley wine is known as Highland barley distillers’ grains. It is sometimes referred to as meal or wine spirits lees. Compared to typical sorghum lees, Highland barley distillers’ grains have a greater protein level of up to 23.57% [5] and a higher gliadin concentration of 21.04% [6]. However, the development and application of β-glucan is the main focus of current research on highland barley distillers’ grains. There is currently little research on gliadin from highland barley distillers’ grains (HBDGG).

Gliadin, primarily derived from various plants or their byproducts, offers several advantages over animal proteins, including biodegradability, biocompatibility, lower cost, and simpler production. Furthermore, they possess the capability to adsorb or covalently attach to molecules exhibiting specific properties. Composite nanoparticles fabricated using gliadin demonstrate enhanced water solubility, improved payload retention, and increased gliadin bioavailability [7]. Nevertheless, hydrophobic amino acids, of which gliadin makes up more than half of their amino acid composition, are undesirable for long-term preservation [8]. Gliadin’s poor water solubility restricts its use as well. Cereal gliadin’s function can be altered by enzymatic, chemical, or physical means. To improve their functional qualities, common strategies involve complexing cereal gliadin with protein fractions, polysaccharides, polyphenols, and other active small molecules through covalent or non-covalent binding. By offering superior protection and a delayed release of active components, these intricate systems can increase the stability of single-protein delivery methods [9,10,11]. Han [12] discovered that the composite system exhibited superior storage stability, ionic strength stability, and acid-base stability when the mass ratio of peach gum polysaccharide to maize gliadin was 1:1. Better acid-base stability, ionic strength stability, and storage stability were all exhibited by the composite system.

Chitosan (Cs) is a natural polysaccharide polymer for encapsulating and delivering bioactive ingredients due to its biocompatibility, biodegradability, and non-toxicity [13,14,15]. Deacetylation of chitin by 50% or more yields Cs, also referred to as deacetyl chitin. The chitosan molecule’s amino group may become protonated in an acidic environment, giving it a positive charge [16]. Depending on how they develop, cereal gliadin–polysaccharide composite nanoparticles can be classified as either covalently cross-linked or non-covalently bonded [17]. Because of its low energy consumption, this composite system tends to concentrate more on non-covalent interactions [18]. The foundation of electrostatic complexation is the idea that oppositely charged proteins and polysaccharides are drawn to one another by electrostatic forces. As a result, it does not need energy, heating, or high pressure. When working with proteins that bind to cationic polysaccharides at pH levels above their isoelectric point or to anionic polysaccharides at pH levels below their isoelectric point, this approach is perfect for the self-assembly of PPNPs [19]. Simple preparation conditions, a reversible nanoparticle structure, the lack of harmful cross-linking chemicals, and strong biocompatibility are characteristics of this kind of binding. For example, composite particles with a core–shell structure were produced by combining negatively charged polysaccharides with positively charged zein [20]. Prolamin–polysaccharide complexes have been demonstrated to effectively enhance the stability and water solubility of nanoparticles, serving as effective delivery vehicles to improve the overall physiological efficacy of bioactive compounds. Currently, Cs has been used to coat zein nanoparticles for delivering nutrients [21,22]. For instance, Sha et al. [23] developed chitosan/genipin/zein nanoparticles for the encapsulation and release of curcumin. Pauluk et al. [24] fabricated chitosan-coated zein nanoparticles to deliver resveratrol, where the chitosan coating enhanced the gastrointestinal stability of the encapsulated resveratrol. Li et al. [25] enhanced the stability of curcumin in an in vitro gastrointestinal model by encapsulating it within zein–chitosan composite particles, effectively protecting curcumin from degradation in the gastrointestinal environment. However, currently, the fabrication of HBDGG-Cs composite nanoparticles has received scant attention in the published literature. Building upon these findings, composite systems based on HBDGG and Cs demonstrate significant potential as effective delivery vehicles for enhancing the overall physiological efficacy of bioactive compounds.

The pH-driven approach, solvent evaporation, antisolvent precipitation, and antisolvent co-precipitation are common techniques for creating cereal gliadin–chitosan composite nanoparticles [26]. Since it does not require complicated equipment or operating conditions, the antisolvent precipitation method is one of the most widely used techniques for creating cereal gliadin-based composite nanoparticles.

Based on this research background, this study synthesized HBDGG-Cs composite nanoparticles using the antisolvent precipitation method. The goal was to analyze the interaction mechanism, structural changes, and microscopic morphology of HBDGG-Cs complexes with different mass ratios. Additionally, the stability of HBDGG-Cs_1:1_ composite nanoparticles was investigated to provide a theoretical basis and technology for the application of the HBDGG-Cs active substance delivery system.

## 2. Results and Discussion

### 2.1. Effect of HBDGG/Cs Ratios on Properties of HBDGG-Cs Composite Nanoparticles

**Figure 1:**
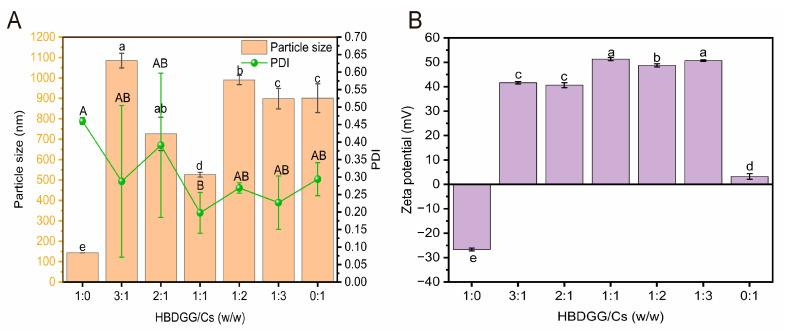
(**A**) Particle size, PDI; (**B**) zeta potential of HBDGG-Cs composite nanoparticles at different mass ratios. Note: Different uppercase and lowercase letters represent significant differences between groups, *p* < 0.05.

As shown in Figure 1A, the hydrated diameter of HBDGG was 143.06 ± 0.01 nm. The hydrated diameters of the HBDGG-Cs composite nanoparticles significantly increased to between 306 and 1084 nm after Cs was added. Their sizes were closely correlated with the HBDGG and Cs mass ratio. When the HBDGG to Cs mass ratio was 3:1 or 2:1, the composite particles had larger hydration diameters of 1084.97 ± 35.83 nm and 1004.97 ± 81.54 nm, respectively, and higher PDI values. This indicated poor dispersion of the system, possibly due to the lower Cs content not completely covering the HBDGG particles. This led to bridged flocculation between the proteins and aggregation of the particles [27]. Li et al. [28] also observed protein flocculation and coagulation in a study related to corn protein and soybean polysaccharides. As the content of Cs increased, the surface charge progressively increased. This enhancement in electrostatic repulsion between composite particles consequently resulted in a reduction in particle size [29]. When the HBDGG: Cs mass ratio was 1:1, the hydrated diameter was 526.10 ± 11.78 nm, and the PDI decreased. This indicates that the homogeneity and stability of the composite particle solution improved at this ratio.

The greater the absolute value of the potential, the more stable the solution [30]. As shown in Figure 1B, the HBDGG particles had a negative potential of −26.67 ± 0.58 mV, and Cs exhibited a positive zeta potential of +3.22 ± 1.14 mV. After Cs was added, the surface of the composite particles became positively charged, indicating that Cs was effectively adsorbed onto the HBDGG surface. A stable complex formed between the HBDGG and Cs through electrostatic and non-electrostatic interactions. As the Cs content increased, the surface charge of the samples increased gradually, enhancing the electrostatic adsorption and making the nanoparticles more compact. When the HBDGG: Cs mass ratio was 1:1, the nanoparticles had the smallest hydrated diameter, and the PDI reached its minimum value (0.20 ± 0.06). At this ratio, the zeta potential was 51.31 ± 0.66 mV. This indicates that the HBDGG-Cs_1:1_ nanoparticle dispersion and stability were optimal. This may be due to the formation of a well-encapsulated shell–nucleus structure of the complexes, which makes the system more stable [31].

As the Cs content increased to a HBDGG-Cs mass ratio of 1:2 and 1:3, the hydrated diameters of the complexes increased to 990.27 ± 22.87 nm and 898.33 ± 50.25 nm, respectively. This increase may be attributed to excess Cs molecules cross-linking with each other, leading to inter-particle aggregation and an increase in the hydrated diameter of the composite particles. Wang et al. [32] reported similar results when using ovalbumin and pectin.

In summary, based on the comprehensive analysis of particle size, PDI, and zeta potential, the complexes prepared at an HBDGG-Cs mass ratio of 1:1 exhibited the smallest particle size and the most homogeneous and stable solution. The HBDGG-Cs_1:1_ complex is therefore a suitable candidate for subsequent studies on HBDGG-Cs nanoparticle stability.

### 2.2. Structural Properties

#### 2.2.1. Ultraviolet–Visible Spectrum

**Figure 2:**
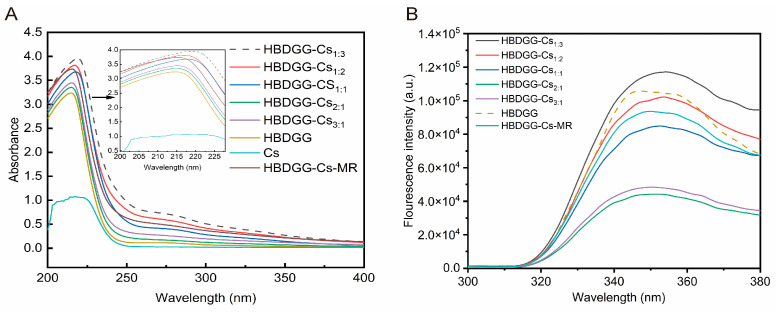
(**A**) UV-Vis spectra; (**B**) intrinsic fluorescence emission spectra of HBDGG, Cs, and HBDGG-Cs composite nanoparticles at different mass ratios, and conjugates of HBDGG and Cs (HBDGG-Cs-MR).

Changes in the tertiary and quaternary structure of a protein can be reflected to some extent by measuring its UV absorption spectrum. As shown in Figure 2A, HBDGG, like most proteins, exhibits a distinct absorption peak at 280 nm. This peak may be caused by the transition of aromatic rings of tryptophan and tyrosine residues from π to π* in the polypeptide chain [33]. Cs exhibits a maximum absorption peak at 217 nm. The absorption peaks of the composite nanoparticles differed significantly from those of HBDGG. The UV absorbance values of HBDGG-Cs composite particle solutions with different mass ratios increased significantly from the absorption peak intensity. This may be due to the conformational change of HBDGG during its interaction with Cs in the self-assembly process. This interaction causes the unfolding of the protein peptide chain. This results in the destruction of secondary and tertiary structures, exposing the chromogenic groups therein. Thus, the UV absorbance intensities of proteins are elevated [34]. Moreover, the UV absorption peak of the HBDGG composite nanoparticles increased with the addition of Cs, indicating that the Cs altered the molecular structure of the HBDGG, exposing more Trp and Tyr residues. This trend increased with the addition of more Cs.

Compared to HBDGG, the maximum absorption peak wavelength shifted to the red after the reaction of HBDGG with Cs, indicating a change in the microenvironment of the amino acid residues. The covalent cross-linking of HBDGG with Cs unfolded the protein, exposing tryptophan and tyrosine residues to the protein’s surface. This resulted in an increased UV absorbance value [35].

#### 2.2.2. Fluorescence Spectroscopy

As shown in Figure 2B, the maximum fluorescence intensity values for each group of samples occur at wavelengths greater than 330 nm. This indicates that most of the tryptophan residues (Try) in the samples are in a polar environment outside of the protein molecule [36]. In the absence of Cs^+^, HBDGG exhibited maximum emission intensity at 347 nm when excited at 280 nm. When Cs was added, the fluorescence intensity of HBDGG decreased. With an increase in Cs content, the fluorescence intensity increased, and the maximum fluorescence emission wavelength red-shifted from 350.00 nm at a 3:1 ratio to 354.00 nm at a 1:3 ratio. Cs complexation caused an endogenous fluorescence burst of HBDGG, indicating that Cs interacted with HBDGG and folded its protein structure to some extent, increasing the hydrophobicity of the microenvironment in which the amino acids of HBDGG’s fluorescent chromophore were located. This indirectly indicates that Cs is bound to HBDGG through hydrophobic interactions.

The HBDGG fluorescence intensity gradually weakened with decreasing Cs addition. This was attributed to Cs’s spatial site-blocking effect, which inhibited macroscopic over-aggregation of HBDGG. This effect became more obvious with increasing Cs addition. Due to the interaction between the HBDGG and Cs molecules, some tryptophan residues were wrapped inside the macromolecular chain. This disturbed the microenvironment of the amino acid due to the repulsive force between the HBDGG molecules and the electrostatic attraction between the HBDGG and Cs molecules. This resulted in the movement of the tryptophan residues into a more hydrophobic environment. However, when there was an excess of Cs in the complex system (a mass ratio of 1:3), the peak fluorescence intensity of the HBDGG increased. This indicates an increase in polarity and a decrease in the hydrophobicity of the microenvironment surrounding the fluorescent moiety within the protein. Compared to HBDGG, the fluorescence intensity of HBDGG decreased after the metal reaction with Cs. This phenomenon is attributed to the shielding effect of the polysaccharide chain on the protein’s tryptophan residue [37]. The maximum absorption peak of tryptophan fluorescence exhibited a significant red shift, indicating that covalent binding altered the tertiary structure of the protein. This suggests that tryptophan shifts to a more hydrophilic environment due to the introduction of hydrophilic hydroxyl and carboxyl groups by Cs [38].

The remaining groups of samples exhibited reduced fluorescence intensity compared to HBDGG. The HBDGG-Cs covalent complex had a greater reduction in fluorescence intensity than the HBDGG-Cs nanocomposite particles when the HBDGG-Cs ratio was 1:2. The presence of polysaccharide chains may have shielded the tryptophan residues of the proteins. Additionally, the covalent binding of sugar chains to proteins may have a stronger shielding effect than non-covalent binding [39]. This result is consistent with the findings of Xue et al. [40].

#### 2.2.3. Infrared Absorption Intensity

**Figure 3:**
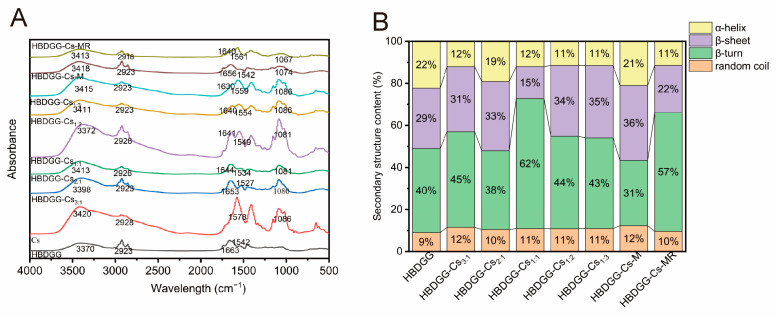
(**A**) FT-IR spectra; (**B**) second structure content of HBDGG, Cs, and HBDGG-Cs composite nanoparticles at different mass ratios, mixtures, and conjugates of HBDGG and Cs.

FTIR spectra reflect information about the characteristic groups in compounds and can be used to study intermolecular interaction forces between Cs and HBDGG in nanoparticles. The FTIR spectra of Cs, HBDGG, HBDGG-Cs-M, HBDGG-Cs-MR, and composite nanoparticles with different HBDGG-Cs mass ratios are shown in Figure 3A. The three main characteristic peaks of HBDGG are located at 3370 cm^−1^ (3100–3600 cm^−1^), 1663 cm^−1^ (1700–1600 cm^−1^), and 1542 cm^−1^ (1500–1600 cm^−1^). These peaks are related to the O-H stretching vibration, the amide I band, and the amide II band, respectively. The amide I band is related to the stretching of the C=O group. The absorption peaks of the amide II band are mainly caused by the bending vibration of C-N-H and the stretching vibration of C-N and C-C [41].

The addition of Cs caused a change in the characteristic peak of HBDGG, shifting the hydroxyl group stretching vibration peak between HBDGG and Cs. As the Cs content increased, the position of the characteristic peak of the HBDGG-Cs composite nanoparticles moved closer to Cs, suggesting the formation of hydrogen bonds between the amide group of HBDGG and the hydroxyl group of Cs [42]. This result is in agreement with the findings of Li et al. [43].

Similarly, adding Cs causes changes in the β-sheet structure, inducing displacements in the amide I and amide II bands of the HBDGG-Cs composite nanoparticles. This suggests a spatial reconfiguration of the HBDGG, exposing more hydrophobic clusters and enhancing hydrophobic interactions with Cs [44]. It was found that the amide I and amide II peaks were lower than those of HBDGG when the HBDGG-Cs mass ratios were 3:1, 2:1, and 1:1. This further indicated the presence of hydrophobic interactions between the HBDGG and Cs molecules. These results are consistent with those of Chen et al. [45] and prove the existence of hydrophobic interactions. The peaks of amide I and amide II were found to be lower than those of HBDGG when the mass ratio of HBDGG to Cs was 3:1, 2:1, or 1:1. These results further suggest the existence of hydrophobic interactions between HBDGG and Cs molecules. These results align with the findings of Wu et al. [46], which prove the existence of these interactions. As can be seen in the figure, both Cs and the HBDGG-Cs composite nanoparticles contain characteristic peaks near 1081 cm^−1^, whereas no peaks appear for HBDGG at this wavelength. The increase in peak area with the amount of Cs suggests that the HBDGG particles are encapsulated by Cs. There are hydrogen-bonding, electrostatic, and hydrophobic interactions between HBDGG and Cs, as well as the formation of HBDGG composite particles through the encapsulation of HBDGG particles in Cs. The HBDGG particles form HBDGG-Cs composite nanoparticles.

The O-H stretching vibration peaks of the HBDGG-Cs glycosylation products and physical mixtures are significantly broader than those of HBDGG. This may be due to the stretching vibrations of the N-H and O-H bonds in intermolecular hydrogen bonding [47]. The new peak at 1086 cm^−1^ can be attributed to the C-O-C stretching vibration. The absorption peaks at 1000–1180 cm^−1^ are the C-O-C stretching vibration peaks [48]. The samples containing polysaccharides exhibited clear absorption peaks, and the peak intensity of the HBDGG-Cs glycosylation product weakened, indicating the glycosylation reaction of barley lees alcohol-soluble protein with chitosan.

The absorption bands of the HBDGG-Cs glycosylation products shifted compared to HBDGP: they shifted to 1640 cm^−1^ at 1663 cm^−1^ and to 1561 cm^−1^ at 1542 cm^−1^. These shifts correspond to amide II (N-H bending vibration) and can be attributed to the Maillard reaction [49]. The intensity of the corresponding peaks was also reduced, indicating that the Maillard reaction of HBDGG with Cs produces an Amadori mixture (C=O), a Schiff base (C=N), and a pyrazine (C-N). These compounds change the protein conformation, and heating promotes this change [50]. This result is in agreement with the findings of Ye et al. [51].

#### 2.2.4. Protein Secondary Structure Content

As shown in Figure 3B, the α-helix content decreased from 22% to 11%, the β-sheet content increased from 29% to 35%, the β-turn content increased from 40% to 62%, and the irregular curl content increased from 9% to 12% after Cs complexed with HBDGG. These results are consistent with those reported by [52]. This phenomenon may be due to the partial unfolding of the protein resulting from electrostatic interactions between HBDGG and Cs. These changes primarily occur at the level of the protein’s ordered secondary structure [53]. In the HBDGG-Cs complex, the β-sheet content of the protein increased, and the amide I band shifted, which thus confirms the dominant role of hydrogen bonding in complex formation.

After the mixing or covalent binding of Cs, the α-helix content in HBDGG decreased significantly, while the irregular coil content increased to varying degrees. This suggests that binding polysaccharides alters the original conformation of HBDGG [54]. Significant changes in secondary structure indicate that covalent interactions between proteins and polysaccharides occur through the Maillard reaction. This reaction involves the interaction of biomolecules and the thermal denaturation of proteins in the primary stage. In this stage, the protein structure unfolds, and the ε-amino group in the α-region of the peptide chain covalently binds to the carbonyl group in the polysaccharide [55].

#### 2.2.5. Analysis of X-Ray Diffraction (XRD) 

**Figure 4:**
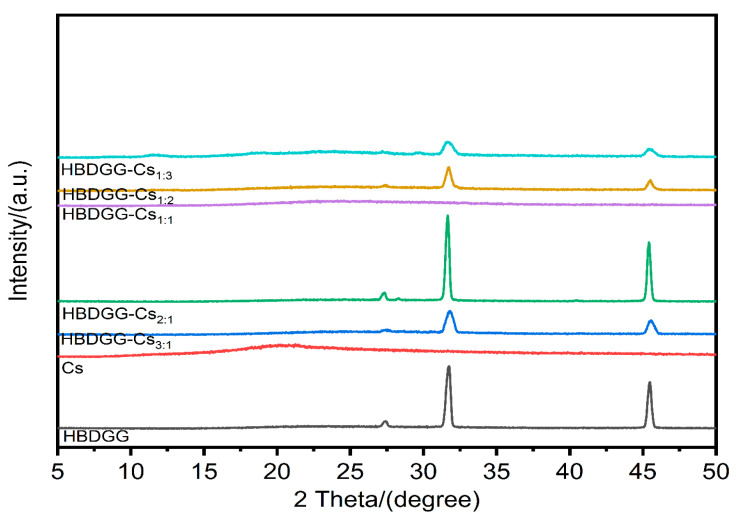
X-ray diffraction of HBDGG, Cs, and HBDGG-Cs composite nanoparticles at different mass ratios.

X-ray powder diffraction (XRD) analyzes the intermolecular interactions of particles and reflects their crystal structure [56]. As shown in Figure 4, HBDGG shows a narrower diffraction peak at 2θ of 25.50°, in addition to two broader diffraction peaks in the region of 31.72° and 45.48°, indicating the amorphous structural properties of HBDGG [57].

The presence of Cs in the crystalline structure is shown by its large peak at 20.17°. X-ray diffraction spectra of HBDGG-Cs composite particles at various mass ratios reveal the broad peak of Cs disappearing and the characteristic diffraction peaks of HBDGG becoming less intense. This implies that the nanoparticles are amorphous and that the electrostatic interactions and hydrogen bonding between HBDGG and Cs take place inside the nanoparticle matrix.

#### 2.2.6. Microstructure Analysis

**Figure 5:**
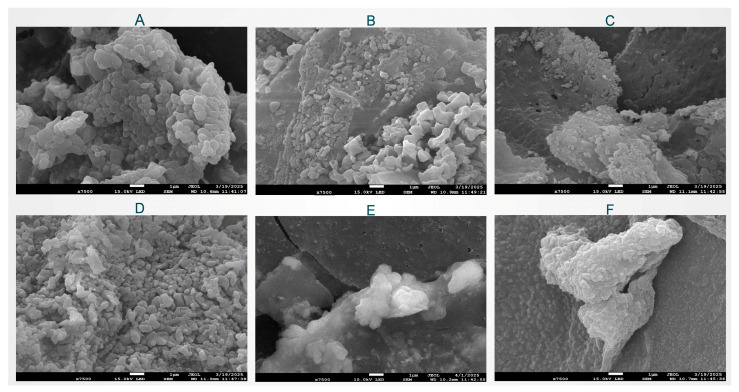
Scanning electron microscopy of HBDGG-Cs composite nanoparticles at different mass ratios: (**A**) HBDGG-Cs_1:0_; (**B**) HBDGG-Cs_3:1_; (**C**) HBDGG-Cs_2:1_; (**D**) HBDGG-Cs_1:1_; (**E**) HBDGG-Cs_1:2_; (**F**) HBDGG-Cs_1:3_.

As shown in Figure 5A–F, both single HBDGG particles and HBDGG-Cs nanoparticles exhibit typical spherical structures, regardless of the mass ratio. Compared to single HBDGG particles, HBDGG-Cs nanoparticles form larger aggregates.

The particle size grew and gaps between the aggregates emerged when the HBDGG-Cs mass ratio was 3:1, indicating that the addition of Cs changed the system’s highly hydrophobic environment and facilitated the dispersion of the composite particles. The nanoparticles formed a tight network structure that was independent of one another when the mass ratio of HBDGG to Cs was 1:1. Higher Cs additions resulted in closely packed nanoparticles (HBDGG-Cs mass ratios of 1:2 and 1:3). This might be explained by interactions between proteins and polysaccharides that aided in particle adherence as well as by extra polysaccharide molecules wrapping around the surfaces of the HBDGG particles.

### 2.3. Stability Analysis of HBDGG-Cs Nanoparticles

#### 2.3.1. Storage Stability

**Figure 6:**
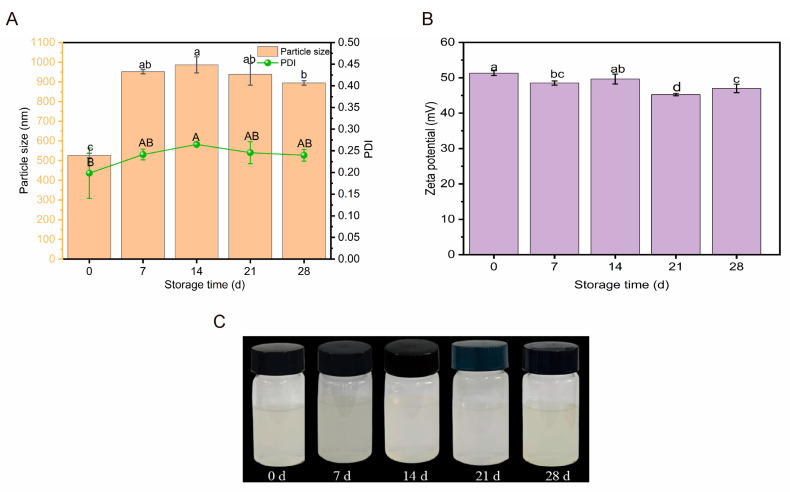
(**A**) Particle size, PDI; (**B**) zeta potential; (**C**) appearance of HBDGG-Cs composite nanoparticles under differentstorage times. Note: Different uppercase and lowercase letters represent significant differences between groups, *p* < 0.05.

As shown in Figure 6A,C, the hydrated diameter of the HBDGG-Cs nanoparticles increased with storage time, and the PDI increased and then decreased. During the first seven days of storage, the particle size of HBDGG-Cs_1:1_ increased from 526.10 ± 11.78 nm to 951.70 ± 10.54 nm. The PDI value increased from 0.19 ± 0.06 to 0.24 ± 0.01. This increase may be due to Brownian motion triggering collisions between particles, leading to slight aggregation. During the final stage of storage (7 to 28 days), the changes in particle size and PDI stabilized, and no significant differences were observed (*p* > 0.05). This indicates that the aggregation process had essentially ceased. As shown in Figure 6B, the electric potential decreased overall during storage, indicating that electrostatic adsorption between the complexes weakened with increased storage time. Therefore, HBDGG-Cs_1:1_ composite nanoparticles exhibit good storage stability at 4 °C. Their particle size, PDI, and electric potential values stabilize at the late stage of storage, suggesting that the complex system can maintain a stable physical morphology under these conditions.

#### 2.3.2. Ionic Stability

**Figure 7:**
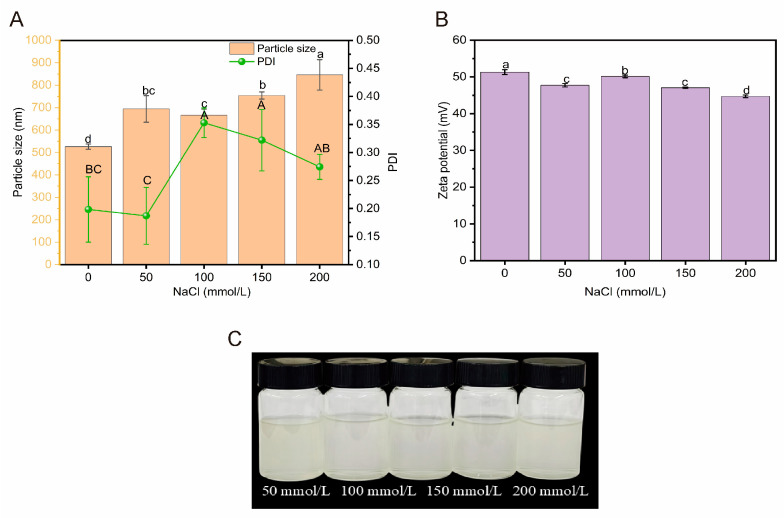
(**A**) Particle size, PDI; (**B**) zeta potential; (**C**) appearance of HBDGG-Cs composite nanoparticles at different NaCl concentrations. Note: Different uppercase and lowercase letters represent significant differences between groups, *p* < 0.05.

As shown in Figure 7A–C, the hydrated diameter of HBDGG-Cs_1:1_ composite nanoparticles significantly increased from 526.10 ± 11.78 nm to 694.30 ± 59.58 nm (*p* < 0.05) as the NaCl concentration increased from 0 to 50 mmol/L. The electric charge significantly decreased from 51.30 ± 0.67 mV to 47.73 ± 0.46 mV (*p* < 0.05). This was due to the neutralization of the surface charge of the complexes by the Na^+^ and Cl^−^ ions of NaCl, which resulted in weaker electrostatic adsorption between the particles and an increase in particle size.

The hydrated diameter of HBDGG-Cs_1:1_ decreased from 694.30 ± 59.58 nm to 666.03 ± 27.10 nm when the NaCl concentration was between 50 and 100 mmol/L. This demonstrated how the salting-out effect progressively took hold as the concentration of NaCl rose. Water molecules on the nanoparticles’ surface were dehydrated at this concentration by NaCl. The particles’ binding became closer as a result. Salting out is the term for this procedure. The HBDGG-Cs_1:1_ composite nanoparticles’ hydrated diameter rose dramatically to 845.96 ± 67.80 nm when the NaCl concentration hit 200 mmol/L. This increase could be explained by the intense electro-neutralization brought on by the high concentration of Na+ and Cl+ ions, which caused a notable shift in the particles’ surface charge. Particle size increased as a result of the complexes’ surface charge dropping to 44.72 ± 0.35 mV and an increase in particle aggregation [58,59]. The findings of this investigation align with Zhang’s findings [60].

#### 2.3.3. Acid-Base Stability

**Figure 8:**
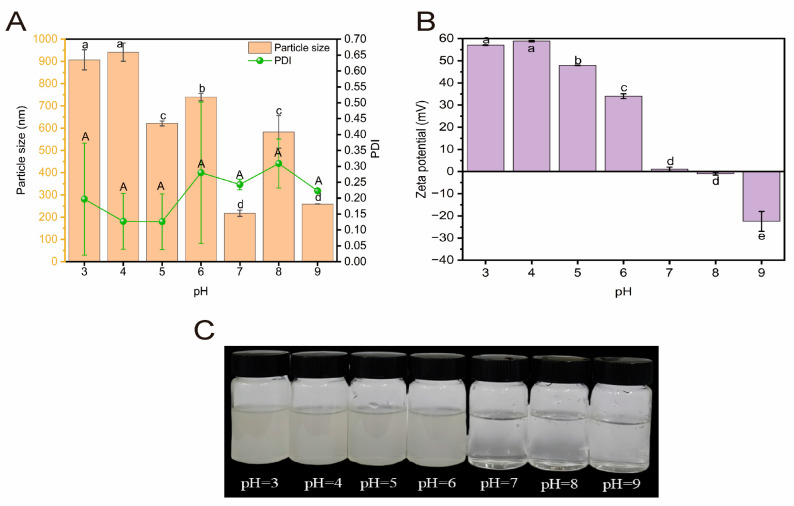
(**A**) Particle size, PDI; (**B**) zeta potential; (**C**) appearance of HBDGG-Cs composite nanoparticles within different pH ranges. Note: Different uppercase and lowercase letters represent significant differences between groups, *p* < 0.05.

Changes in the alkaline environment affect the size and uniformity of the hydration diameter of HBDGG-Cs_1:1_ composite nanoparticles, as well as their surface charge. As shown in Figure 8A,B, the hydration diameter of the nanoparticles decreases overall in the pH range of 3–9. In the pH range of 3–4, the hydration diameter of the particles did not change significantly. When the pH was 7, the hydration diameter decreased significantly to a minimum value of 217.8 ± 14.15 nm. At a pH of 5, the PDI of the HBDGG-Cs_1:1_ complex was smaller (0.13 ± 0.09), indicating a more homogeneous particle distribution. The PDI of HBDGG-Cs_1:1_ composite nanoparticles was smaller at this time (0.13 ± 0.09), suggesting a more uniform particle distribution. At pH 8, the PDI reached a maximum value of 0.31 ± 0.08; however, at other pH levels, the PDI was less than 0.30. Thus, the HBDGG-Cs_1:1_ complex exhibited better dispersion and uniformity within the pH range of 3–9.

As can be seen in Figure 8C, in the pH range of 3–4, which is below the isoelectric point of 4.0, the HBDGG is positively charged. In acidic conditions, the Cs is also positively charged. The surface charge of the HBDGG-Cs_1:1_ composite nanoparticles is positive. At this pH range, electrostatic repulsion dominates between HBDGG and Cs. In the pH range of 4–7, HBDGG is negatively charged, and Cs is positively charged in acidic conditions. The surface charge of HBDGG-Cs_1:1_ composite nanoparticles is positive, which may be caused by the negative charge in HBDGG interacting with Cs molecules through electrostatic attraction. In the pH range of 7–9, the absolute value of the negative charge carried by the HBDGG-Cs1:1 composite particles increases. Since alcohol-soluble proteins are negatively charged at this pH range, and Cs is also negatively charged under alkaline conditions, electrostatic repulsion dominates between HBDGG and Cs. However, the electrostatic repulsion generated is not strong enough to cause the composite particles to disperse into two phases due to the formation of a core–shell structure, with Cs encapsulating HBDGG [61]. Additionally, it was found that the surface charge of the HBDGG-Cs_1:1_ composite nanoparticles reached 47.86 ± 0.12 mV at pH = 5. Therefore, HBDGG-Cs_1:1_ composite nanoparticles are most stable under acidic conditions (pH = 4–5), showing a smaller particle size, PDI, and larger potential.

## 3. Materials and Methods

### 3.1. Materials

Highland barley distillers’grains were obtained from the Tianyoude Barley Wine Co., Ltd. (Qinghai, China). HBDGG was prepared by the laboratory of the research group. Cs (a deacetylation degree of >95% and a viscosity ranging from 100 to 200 mPa) was commercially purchased from Shanghai Aladdin Biochemical Technology Co., Ltd. (Shanghai, China). All the other chemicals and solvents were of analytical grade and purchased from Qinghai Reiner Biotechnology Co., Ltd. (Qinghai, China)

### 3.2. Extraction of HBDGG

Following a protocol adapted from Guo et al. [62], Highland barley distillers’ grains were dried in a drying oven at 50 ± 0.5 °C for precisely 30 min. Dried Highland barley distillers’ grains were ground in a high-speed grinding mill and passed through a 60-mesh sieve. Subsequently, 200 g of the powder sample was dissolved in 600 mL of an 80% ethanol solution. Samples were magnetically agitated at 500 rpm for exactly 20 min under 25 ± 2 °C using a magnetic stirrer (Shanghai Mowei Biotechnology Co., Ltd, Shanghai, China). Ultrasonication was performed at an ultrasonic power of 400 W and a temperature of 50 °C for 120 min. Post-sonication samples underwent centrifugation (8000× *g*, 20 min, 4 °C) in a high-speed refrigerated centrifuge. The supernatant was dialyzed against ultrapure water in the dark (12 kDa, 48 h, 4 °C) to remove low-molecular-weight impurities. The retentate was concentrated by rotary evaporation under reduced pressure (40 °C, 60 rpm) to eliminate residual ethanol and water. Subsequently, the samples were freeze-dried for 48 h to constant weight using a vacuum freeze dryer (Xiniu Technology Co., Ltd, Zhejiang, China) with the condenser temperature maintained at −53 °C.

### 3.3. Preparation of HBDGG-Cs Nanoparticles

The preparation of HBDGG-Cs nanoparticles was based on the methods of Shen et al. [44]. In brief, HBDGG (0.3 g) was dissolved in an 80% ethanol–water solution with stirring at 750 rpm for 1 h at 25 °C to ensure adequate dissolution. Subsequently, Cs (1.0%, *w*/*v*) was dispersed in glacial acetic acid (1.0%, *v*/*v*) with stirring at 600 rpm overnight to ensure dissolution at room temperature. The particle suspension was then prepared by adding different volumes (10.0, 15.0, 30.0, 60.0, and 90.0 mL) of Cs solution drop-wise to 100 mL of HBDGG solution and stirring at 600 rpm for 1 h, resulting in final mass ratios of HBDGG to Cs of 3:1, 2:1, 1:1, 1:2, and 1:3. The pH was precisely adjusted to 4.25 with 1.0 M NaOH or 1.0 M HCl. The dispersion obtained by injecting HBDGG (0.3%, *w*/*v*) into pure water was set as the control group. The dispersion was stirred for 1 h. Then, ethanol and water were removed from the dispersion in a 40 °C water bath using a rotary evaporator (Zhengzhou Changcheng Technology Industry and Trade Co., Ltd, Zhengzhou, China). Some of the liquid samples were refrigerated at 4 °C for property determination, and the rest of the liquid samples were pre-frozen in the freezer at −80 °C for 12 h, and then vacuum freeze-dried (−50 °C) for 48 h. The freeze-dried samples were placed in a desiccator for spare parts.

### 3.4. Preparation of the HBDGG-Cs Conjugate (HBDGG-Cs-MR)

The preparation of HBDGG-Cs-MR was based on Yang et al.’s method and was appropriately modified [63]. Using a mass ratio of 1:1 between HBDGG and Cs, 0.3 g of HBDGG was dissolved in 100 mL of an aqueous solution with a pH of 13. We added 6 mL of the chitosan solution in an equivalent amount, stirring well to dissolve. The solution had a pH of 13. It was then sealed and heated to 90 °C in a 120 min reaction. Once the reaction was finished, we allowed it to cool to room temperature and set the pH to 8. The solution was then magnetically stirred for one hour at 500 rpm. HBDGG-Cs-MR was obtained following vacuum lyophilization. HBDGG and Cs were physically mixed in a 1:1 ratio to create the physical mixture (HBDGG-Cs-M).

### 3.5. Characterization of HBDGG, Cs, and HBDGG-Cs Nanocomposite Particles

#### 3.5.1. Particle Size, PDI, and Zeta Potential

The particle size, polydispersity index (PDI), and zeta potential of the particulate dispersion were determined using a Nano-Zetasizer nanoparticle size potentiostat (Ltd., Malvern, Worcestershire, UK) according to the described method [64].

The prepared particle dispersions (1 mL) were diluted tenfold to determine the above indices. The samples were equilibrated in the instrument for 120 s before measurement to avoid multiple scattering effects. All samples were measured at 25 °C, and each sample was measured three times, with the average value taken.

#### 3.5.2. UV-Absorbed Intensity

Following the method by Xia [1], the protein concentrations of HBDGG, Cs, HBDGG-Cs-MR, and HBDGG-Cs complexes at various mass ratios were diluted to 0.1 mg/mL with glacial acetic acid (*v*/*v*). The spectra were then scanned from 200 to 400 nm using a UV spectrophotometer (UV-2600i; Shimadzu, Kyoto, Japan) at a medium scanning speed with a bandwidth of 1.0 nm.

#### 3.5.3. Endogenous Fluorescence Spectroscopy

The HBDGG and HBDGG-Cs composite nanoparticles, as well as the HBDGG-Cs-MR, were dissolved according to the method described in Section 3.5.2. The final concentration was determined to be 0.1 mg/mL, and their endogenous fluorescence spectra were measured at room temperature using a fluorescence spectrophotometer (RF-6000; Shimadzu, Kyoto, Japan). The excitation wavelength was set to 280 nm, the slit width was set to 5 nm, and the fluorescence spectra were scanned within the 300–380 nm emission wavelength range.

#### 3.5.4. Fourier Transform Infrared (FT-IR) Spectroscopy

According to the method of Feng et al. [65], the interactions between HBDGG and Cs were analyzed by Fourier transform infrared spectroscopy (FTIR) (Nicolet™ iS50 FTIR; Thermo Fisher Scientific, Waltham, MA, USA). The lyophilized samples of HBDGG, Cs, HBDGG-Cs-MR, HBDGG-Cs-M, and HBDGG-Cs complexes at different mass ratios were accurately weighed, mixed with potassium bromide powder (1:100), ground into a powder using a mortar and pestle, pressed into homogeneous, translucent thin films using a tableting machine (Next day, New Materials Co., Ltd, Jiangsu, China), and finally measured using FTIR. Wave numbers were collected in the range of 4000–400 cm^−1^ with a resolution of 4 cm^−1^.

#### 3.5.5. Analysis of the Secondary Structure

Peak fit v4.12 was used to perform Gaussian deconvolution of the base and second-order derivation of 1700–1600 cm^−1^ in the samples’ infrared spectral findings (SeaSolve Software, Inc., Columbia, MD, USA).

#### 3.5.6. X-Ray Diffraction (XRD)

Lyophilized samples of HBDGG, Cs, and HBDGG-Cs complexes with different mass ratios were weighed accurately according to the described method [66] and examined using an X-ray diffractometer (D-max 2500PC; Ltd., Rigaku, Japan) with a Cu target, set to 40 kV and 25 mA. The scanning range was set to 5–50° with a scanning speed of 4. The step size was 0.05°.

#### 3.5.7. Microstructure

The microstructure of HBDGG-Cs nanoparticles with different mass ratios was observed using field emission scanning electron microscopy (JSM-7900F; JEOL, Ltd, Tokyo, Japan), as described by Hasankhan et al. [67]. The lyophilized samples were sprayed with gold on their surfaces before observation to avoid charging under the electron beam, and then the microstructural images of the samples were acquired at an accelerating voltage of 5 kV.

### 3.6. Stability Analysis of HBDGG-Cs Complexes

#### 3.6.1. Analysis of Storage Stability

A 1:1 HBDGG-Cs complex solution was prepared with a HBDGG mass concentration of 3 mg/mL by using the method described by Han et al. [12]. The solution was stored at 4 °C for 28 days, and the average particle size, zeta potential, and PDI of the solution were determined at days 0, 7, 14, 21, and 28, respectively. Throughout the storage period, the composite nanoparticle dispersion exhibits minimal fluctuations in particle size and polydispersity index (PDI).

#### 3.6.2. Analysis of Ionic Stability

According to the method of Feng et al. [68], a 1:1 HBDGG-Cs complex solution was prepared using 3 mg/mL HBDGG and mixed with NaCl at concentrations of 0, 50, 100, 150, and 200 mmol/L in equal volumes. The mixture was stirred thoroughly and left at 25 °C for three hours.

The solution was then centrifuged at 10,000 rpm for ten minutes to remove the precipitate. After centrifugation and overnight storage at 4 °C, the average particle size, zeta potential, and PDI of the solution were determined. At suitable NaCl concentrations, the composite nanoparticle dispersion maintains a small particle size and a high absolute zeta potential, signifying enhanced stability.

#### 3.6.3. Analysis of Acid-Base Stability

A 1:1 HBDGG-Cs complex solution was prepared with a HBDGG mass concentration of 3 mg/mL by using the method described by Han et al. [12]. The pH value of the sample solution was then adjusted to 4, 5, 6, 7, and 8 using 1 mol/L HCl and NaOH, respectively. The solution was stirred thoroughly, allowed to stand at 25 °C for three hours, and then centrifuged at 10,000 rpm for ten minutes to remove the precipitate. After overnight storage at 4 °C, the average particle size, zeta potential, and polydispersity index (PDI) of the centrifuged solution were determined. Within the optimal pH range, the composite nanoparticle dispersion demonstrates minimal particle size, low polydispersity index (PDI), and a high absolute zeta potential, indicating improved stability.

### 3.7. Statistical Analysis

Data are reported as mean ± standard deviation (Mean ± SD) in triplicate. Statistical analysis was performed using SPSS 27 software (SPSS Inc., USA). Depending on the specific experimental design and data characteristics, data were analyzed using one-way analysis of variance (ANOVA). The criterion for statistical significance was set at *p* < 0.05. Error bars: SD. Data visualization was performed using Origin 2021 software.

## 4. Conclusions

The present study investigated the effect of Cs addition on the properties of HBDGG-Cs nanoparticles prepared by the antisolvent precipitation method. Various analytical means were used to study the effects on particle size, potential, protein structure, microscopic morphology, and stability. The best dispersion and stability of the prepared nanoparticles were achieved at a 1:1 mass ratio of HBDGG-Cs. The introduction of Cs increased the UV absorption intensity and decreased the fluorescence intensity of the complexes, as well as partially unfolding their protein secondary structure. In summary, HBDGG binds to Cs through hydrogen bonding, electrostatic interactions, and hydrophobic interactions. These interactions change the structure and function of HBDGG, thereby improving the stability of the nanoparticles. At the same time, HBDGG binds to Cs through covalent interactions, undergoing a Maillard reaction. Further research is needed to apply the complexes to food and drug applications. This study establishes a theoretical foundation for expanding the applications of HBDGG-Cs complexes in the food industry.

## Data Availability

All relevant data from this study are included in this published article.
